# Ohio State Veterinary Medical Association

**Published:** 1893-11

**Authors:** 


### OHIO STATE VETERINARY MEDICAL ASSOCIATION.

The tenth semi-annual meeting of the Ohio State Veterinary Medical Association was held at Columbus, Ohio, on August 30th and 31st, 1893.

*First Day's Session, August 30th.*

The President, Dr. W. E. Wight in the chair; Dr. W. H. Gribble, Secretary. The minutes of the previous meeting were read and approved. On roll call some eighteen or more members responded.

*Dr. W. T. Davis*, of Maryville, vouched for by Drs. Bull and Gribble was elected to membership.

*Dr W. G. Jones* forwarded a letter of withdrawal from the Association. After discussion, and upon information furnished by Dr. N. Jones, the Secretary was instructed to write to Dr. W. G. Jones and request him to reconsider his resignation.

*J. C. Meyer, Sr.*, reported a peculiar case of Azoturia, and the etiology and pathology of this disease were discussed at length by Drs. Hillock, Cotten and Jones. Adjourned.

*Second Day's Session, August 31st.*

The President, Dr. W. E. Wight, in the chair. Drs. Berry, Dougherty, Dollahunt, and Michener were present as visitors.

*Dr. Collin* reported a case of paraceutesis in a cow, from which by two operations he had removed thereby gallons of serum. The symptoms had been rapid in their appearance but the autopsy showed the lesions of a chronic peritonitis.

*Dr. W. H. Gribble* reported three cases occuring on a farm where the hygienic surroundings were excellent, which presented symptoms as follows: T. 105 degrees F.; pulse rapid and small; respirations short and quick; great debility, especially weakness of the hind legs; appetite, good, almost ravenous throughout; the anus in all cases was dilated, allowing free passage of air into and from the rectum synchronous with each respiratory movement. There was no cough, sore throat, nor discharge from the nostrils. Autopsies were not held. On discussion, Dr. W. E. Wight and Dr. W. H. Gribble considered the trouble as a form of influenza. Others considered it might be malaria, or so-called milk sickness or trembles.

*Dr. Waddle* reported his treatment for tetanus, and *Dr. Carl* gave an account of a fatal case of enlarged larnyx in a cow.

*Dr. Cotten* gave notice that he would propose a radical change in the code of ethics at the next meeting. as the present code is too stringent.

*Dr. W. H. Gribble* moved that a committee be appointed to arrange for an annual banquet at the next annual meeting; seconded by Dr. Cotten; carried.

Adjourned *sine die.*

